# First report of the East African *kdr* mutation in an *Anopheles gambiae* mosquito in Côte d’Ivoire

**DOI:** 10.12688/wellcomeopenres.10662.1

**Published:** 2017-02-09

**Authors:** Mouhamadou Chouaïbou, Fodjo Behi Kouadio, Emmanuel Tia, Luc Djogbenou

**Affiliations:** 1 Centre Suisse de Recherches Scientifiques en Côte d’Ivoire (CSRS), Abidjan, Cote d'Ivoire; 2 Université Nangui Abrogoua, Abidjan, Cote d'Ivoire; 3 Centre d'Entomologie Médicale et Vétérinaire, Université Alassane Ouattara, Abidjan, Cote d'Ivoire; 4 Institut Régional de Santé Publique, Ouidah, Benin; 5 Université d’Abomey-Calavi, Ouidah, Benin

**Keywords:** Malaria, Anopheles gambiae, Vector control, Insecticide resistance, kdr mutation

## Abstract

**Background**. The intensive use of insecticides in public health and agriculture has led to the development of insecticide resistances in malaria vectors across sub-Saharan Africa countries in the last two decades. The *kdr* target site point mutation which is among the best characterised resistance mechanisms seems to be changing its distribution patterns on the African continent. The 1014F *kdr* mutation originally described only in West Africa is spreading to East Africa while the 1014S *kdr* mutation originally described in East Africa, is spreading to West and Central Africa. However, the East- *kdr* mutation has not been reported in Côte d'Ivoire so far.

**Methods**. Immature stages of *Anopheles gambiae s.l.* were collected from breeding sites at the outskirts of Yamoussoukro, Côte d'Ivoire. Emerging 3–5 day old adult female mosquitoes were tested for susceptibility to deltamethrin 0.05%, malathion 5%, bendiocarb 1% and dichlorodiphenyltrichloroethane (DDT) 4% according to WHO standard procedures. A total of 50 *An. gambiae s.l.* specimens were drawn at random for DNA extraction and identification down to the species level. A subsample of 30 mosquitoes was tested for the East-African *kdr* mutation using a Taqman assay.

**Results**. The tested mosquito population appeared to be strongly resistant to deltamethrin (1.03% mortality), bendiocarb (38.46% mortality) and DDT (0% mortality) with probable resistance observed for malathion (92.47%). Among the 41 mosquitoes that were successfully characterized, *An. coluzzii* was predominant (68.3%) followed by *An. gambiae* *s.s.* (19.5%) and a few hybrids (7.3%). Out of 30 specimens genotyped for East- *kdr*, a single hybrid mosquito appeared to be heterozygous for the mutation.

**Conclusion**. The present study revealed the presence of the East- *kdr* mutation in Côte d’Ivoire for the first time in *An. gambiae* and highlights the urgent need to start monitoring the allele and genotype frequencies.

## Introduction

The implementation of malaria control strategies, such as the spraying of residual insecticides and the use of insecticide-treated mosquito nets have led to enormous progress in the control of malaria in Sub-Saharan Africa ( Hemingway, 2014). However, only four classes of insecticides are available for public health purposes, namely pyrethroids, carbamates, organophosphates and organochlorines. Since the discovery of pyrethroids, the only compounds currently recommended for impregnation of mosquito nets owing to their efficacy and safety for humans, no alternative insecticides have been identified. This strong reliance on the same molecules is inevitably translated into a heavy pressure on the target mosquitoes, which consequently have developed resistances to these compounds. The role of the agricultural sector in this selection of resistances remains predominant since anopheline larvae breed in and around farm settings in rural areas. Rice and vegetable cultivation, where 90% of insecticides used are pyrethroids, are of particular concern ( Chouaïbou *et al.,* 2016). Induced resistances may involve increased degradation of the insecticide (so-called metabolic resistance involving three families of enzymes: P-450, esterases and glutathion-S-transferase) ( Hemingway *et al.,* 2004) or a modification of the target preventing the insecticide from reaching its site of action (resistance-based target site point mutation) ( Martinez-Torres *et al.,* 1998; Ranson *et al.,* 2000; Weill *et al.,* 2004).

The *kdr* and *Ace-1* mutations are among the best characterised point mutations. Previous studies aiming at estimating the frequency of the *kdr* mutation and its distribution across the African continent have shown that the 1014F *kdr* mutation first described only in West Africa ( Awolola *et al.,* 2005; Diabate *et al.,* 2004; Tripet *et al.,* 2007; Yawson *et al.,* 2004) has spread to East Africa ( Kulkharni *et al.,* 2006; Ochomo *et al.,* 2015; Verhaeghen *et al.,* 2006). *Vice versa*, the 1014S *kdr* mutation originally described only in East Africa, was observed in West and Central Africa in recent years ( Badolo *et al.,* 2012; Nwane *et al.,* 2011). The underlying resistance mechanism of both East- and West- *kdr* mutation is responsible for cross-resistance to dichlorodiphenyltrichloroethane (DDT) and pyrethroids ( Martinez-Torres *et al.,* 1998; Ranson *et al.,* 2000).

In Côte d’Ivoire resistances to insecticides of four classes used for vector control are prevalent and involve multiple mechanisms (i.e. West- *kdr*, Ace-1, P450s) ( Edi *et al.,* 2014). However, the East- *kdr* mutation has not been reported in this country so far.

## Methods

### Mosquito sampling and susceptibility testing

Immature stages of *An. gambiae s.l.* were collected from breeding sites in October 2015 in rice fields at the outskirts of the city of Yamoussoukro (6°49′13″ N/5°16′36″ W) as part of a large insecticide resistance monitoring study across several cities in Côte d'Ivoire. Larvae sampled from different breeding sites were pooled and allowed to emerge as adults at the insectary of the Centre Suisse de Recherches Scientifiques en Côte d’Ivoire (CSRS). Emerging 3–5 day old adult female mosquitoes were tested for insecticide susceptibility. WHO standard procedures ( WHO, 2013) were followed to monitor the susceptibility of populations to the four chemical groups of insecticides commonly used in public health and agriculture including pyrethroids (deltamethrin 0.05%), organophosphates (malathion 5%), carbamates (bendiocarb 1%) and organochlorines (DDT 4%). Batches of 25 unfed mosquitoes were exposed to insecticide impregnated filter papers obtained from the WHO reference centre at the University Sains Malaysia (Penang, Malaysia). For each test session, 100 mosquitoes (four batches of 25 mosquitoes) were exposed to each insecticide and 50 mosquitoes (two batches of 25 mosquitoes) were exposed to untreated filter papers to serve as controls. The same procedure was carried out using reference susceptible *An. gambiae* mosquitoes (Kisumu strain) obtained from Liverpool School of Tropical Medicine in order to assess the quality of the papers used. All bioassays were conducted at insectary conditions (temperature of 25–27°C and 70–90% relative humidity) and WHO criteria were used as a guideline to assess the phenotypic resistance status of the tested mosquito populations ( WHO, 2013). According to those criteria, resistance is demonstrated by mortality rates <90%, 90–98% suggests increased tolerance (resistance has to be confirmed) and mortality rates >98% are indicative of susceptibility.

### Molecular identification and *kdr* genotyping

For molecular identification, 50 *An. gambiae s.l.* specimens were drawn at random from the pool of unexposed mosquitoes (bioassay controls) for DNA extraction. Individual mosquitoes were identified down to their species level using the Sine-PCR method ( Santolamazza *et al.,* 2008). Among these, 30 mosquitoes were tested for the East-African *kdr* mutation applying a Taqman assay ( Bass *et al.,* 2010). The reaction was performed using an Agilent Stratagene MX3005 qPCR thermal cycler in 10 μl final volume containing SensiMix from Bioline, a primer coupled to a probe and water. The cycling conditions used were 10 min at 95°C, 40 cycles of 10 s at 95°C and 45 s at 60°C. Two probes labelled with fluorochromes FAM and HEX were used to detect the mutant allele and the wild type susceptible allele, respectively. Genotypes were scored after real time amplification as dual colour scatter plots produced by the MX3005P v4.10 software.

## Results

### Resistance status

The *An. gambiae* Kisumu reference strain exhibited full susceptibility (100% mortality) to all the insecticide treated papers confirming appropriate quality. Mortality in the control groups was consistently below 5%, and thus did not require any correction. Based on WHO criteria ( WHO, 2013), the wild mosquito population of Yamoussoukro appeared to be strongly resistant to deltamethrin (1.03% mortality), bendiocarb (38.46% mortality) and DDT (0% mortality). A probable resistance was, in addition, seen for malathion with a mortality rate of 92.47%.

### Species identification and *kdr* genotyping

**Figure 1.**
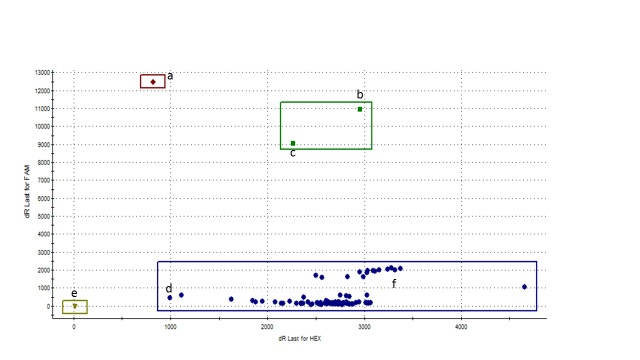
East African *kdr* genotype of wild Yamoussoukro *Anopheles gambiae s.l.* population. The ‘a’, ‘b’, ‘d’ and ‘e’ on the figure are the positive controls respectively for the homozygous mutant allele, heterozygous mutant/susceptible allele, homozygous susceptible allele and blank. One single mosquito (‘c’) displayed the heterozygous mutant/susceptible allele as it appeared to be susceptible for West- *kdr* mutation. All the other samples (‘f’) displayed the susceptible alleles.

Out of the 50 mosquitoes that were used for DNA extraction, the species was successfully characterised in 41. Among these, *An. coluzzii* (former M molecular form) was predominant (n=28; 68.3%) followed by *An. gambiae s.s.* (former S molecular form) (n=8; 19.5%) and a few hybrids (M/S) (n=3; 7.3%). From the 30 specimens genotyped for East- *kdr*, a single hybrid (M/S) mosquito appeared to be heterozygous for the mutation ( Figure 1). Based on the fact that this specimen was heterozygous, it was further characterized for West- *kdr* to verify that it was not a double mutation West-East- *kdr*. However, it appeared to be negative for the West- *kdr* mutation.

## Discussion

At least 64 countries with on-going malaria transmission around the globe have reported resistance to at least one insecticide in at least one vector ( WHO, 2012). In Côte d'Ivoire, resistance to the four families of insecticides was described within the same vector population simultaneously (Behi Fodjo and Mouhamadou Chouaibou, personal communication; Edi *et al.,* 2012) involving several mechanisms at the same time ( Edi *et al.,* 2012). The present study describes the East- *kdr* mutation for the first time in Côte d'Ivoire and delivers further proof for a pan-African propagation of the *kdr* resistance phenomena. The alarming new occurrence of this mutation in Ivorian anopheles mosquito populations represents a major threat to on-going vector control activities as the current strategy of the National Malaria Control Program in the country is heavily based on the use of long lasting nets. According to WHO, with the current level of global vector control coverage, about 220 000 children under the age of five are saved each year ( WHO, 2012); if pyrethroids were to lose their effectiveness due to resistances this number could decrease significantly. Moreover, some laboratory studies have shown that female anopheline mosquitoes with insecticide resistance alleles affect vector competence by increasing susceptibility to plasmodium ( Alout *et al.,* 2013; Ndiath *et al.,* 2014). If the same applies to field populations, then we are facing a potential dramatic increase in malaria transmission. Thus, there is an urgent need to carry out concrete actions for resistance management by using completely new mode of action insecticides and not reformulating insecticides already in use for agriculture. These insecticides could be alternated in time (rotation) and in space (mosaic) or simultaneously (mixture). The rotation and mosaic methods are based on the principles of limiting the duration of exposure of each insecticide to the target. The strategy of mixing insecticides is based on the assumption that the compounds could act in synergy, or that each insecticide in the mixture will be able to eliminate those individuals that are susceptible to it. The Innovative Vector Control Consortium (IVCC) has promised to deliver three new insecticides with totally novel and different modes of action by 2022. Using insecticides with different modes of action in rotation, mixture or mosaic could break the cycle of insecticide resistance and underpin a global malaria elimination and eradication program.

## Conclusion

The present study confirms the presence of the East- *kdr* mutation in Côte d’Ivoire for the first time in *An. gambiae*. Even though the mutation was observed in a single mosquito at the heterozygous state it is of utmost importance to start monitoring the allele and genotype frequencies. This study should further aid the rational planning of insecticide resistance management deployed in Côte d’Ivoire and more widely in Africa.

## Data availability

The data referenced by this article are under copyright with the following copyright statement: Copyright: © 2017 Chouaïbou M et al.

Data from: First report of the East African kdr mutation in an *Anopheles gambiae* mosquito in Côte d’Ivoire DOI: 10.6084/m9.figshare.4564831.v1 (Chouaïbou *et al.*, 2017).
