# Supplementary material for: First report of the East African kdr mutation in an Anopheles gambiae mosquito in Côte d’Ivoire
Source: Wellcome Open Res. 2017 Feb 9;2:8. [Version 1] doi: 10.12688/wellcomeopenres.10662.1 (PMC5351847; doi:10.12688/wellcomeopenres.10662.1)
Supplement: Peer review history

# Peer review: Referee response for version 1

**DOI:** 10.21956/wellcomeopenres.11491.r20078

**Published:** 2017-02-27

**Authors:** Fredros O. Okumu

**Affiliations:** 1 Environmental Health and Ecological Sciences Thematic Group, Ifakara Health Institute (IHI), Ifakara, Tanzania

***Title and Abstract***:

 Yes! The title is appropriate.

***Article content***:

- The design is well explained and can be readily repeated. The approach is great and would be effective for answering the stated question. The contents of the study is sound as well. However, I have picked up one issue around the way the results are interpreted and need for further verification of the results. I would like therefore, to raise the following concern (point (i) below), and some additional comments (points (ii) to (iv) below) for improvement:

1. One would expect that once the first indications of the **East *-kdr*** alleles were observed; and given that this was a single specimen which was heterozygous, the researchers should have conducted an expanded survey in the study area to try and confirm presence of this mutation on the vector populations. Having said that, the fact that **East *-kdr*** mutations have already been observed in other West African countries outside the Cote d’Ivoire, one might argue that it was only a matter of time before it is observed in this current location. Nevertheless, as it stands, this first report is made based on a single specimen, without any additional verification. I assume that the authors intend to conduct such verification and present additional data, possibly in a follow up publication.
2. The labelling and caption for Figure 1 can be confusing for some readers. It requires greater clarity. For example, the use of the term “susceptible” in this figure does not appear mean the same thing as the term “susceptible” as used when describing results of the WHO susceptibility assays.
3. Third (discussion section), in addition to the observations of **East- *kdr***, another significant finding here is the extremely low mortality observed among mosquitoes exposed to pyrethroids (1% with deltamethrin), carbamates (38% with bendiocarb) and organochlorides (0% with DDT). These high resistance levels may seem obvious and commonplace in West Africa, but they do warrant greater discussion, with relevance to current vector control tools. A comment of any results of previous intensity assays done in the area would be useful in this context.
4. Lastly, the authors state in the discussion that “ *According to WHO, with the current level of vector control coverage, about 220,000 children below 5 years are saved each year; if pyrethroids were to lose their effectiveness due to resistances, this number could decrease significantly*”. Well, given the results from this specific study and also a number of other studies from the region, the pyrethroids have already clearly lost their efficacy against mosquitoes. So perhaps an important question is whether and how this change is in any way associated with the overall effectiveness of current interventions.

The authors make a case for further surveillance to monitor east-KDR prevalence in the area. The current report is based on a single specimen, so such additional surveys, including some focus on confirming presence of east-KDR are warranted.

***Data (if applicable)*** *:*

 Enough information has been provided to be able to replicate the experiment. The data is in a usable format/structure and all the data has been provided.

I have read this submission. I believe that I have an appropriate level of expertise to confirm that it is of an acceptable scientific standard.

# Peer review: Referee response for version 1

**DOI:** 10.21956/wellcomeopenres.11491.r20361

**Published:** 2017-02-20

**Authors:** Cyrille Ndo

**Affiliations:** 1 Malaria Research Laboratory, Coordination Organization for the Control of Endemic Diseases in Central Africa (OCEAC), Yaoundé, Cameroon

1. **Title and Abstract: **
  Yes, the title is appropriate for the content of the article and the abstract represents a suitable summary of the work.
2. **Article content: **
  The design, methods and analysis of the results from the study have been clearly explained and they are appropriate for the topic being studied.
3. **Results: **
  Data are well presented and enough information has been provided. Therefore everybody with appropriate scientific background could replicate the experiment.
4. **Discussion and conclusion: **
  According to me the conclusions are sensible, but not totally balanced and justified on the basis of the results of the study.
  In my opinion, the detection of East-kdr in Ivory coast where it has not been reported yet is a proof that resistance genes are spraying across the continent. However, by saying that " *The alarming new occurrence of this mutation in Ivorian anopheles mosquito populations represents a major threat to on-going vector control activities as the current strategy of the National Malaria Control Program in the country is heavily based on the use of long lasting nets*", one could understand or assume that the East-Kdr mutation confers greater resistance than the West-kdr mutation which is already widespread in Ivory Coast. Therefore by identifying this mutation in this country, the level of resistance in *Anopheles* population would increase. Is this the case?
  According to some authors (eg: Reimer et al. 2008) the West-kdr seems to confer greater resistance than the East-kdr. Therefore, I suggest the authors of this paper to add in the discussion information about the real impact of the presence of East-kdr or both mutations, on level of resistance of local malaria vector populations.

I have read this submission. I believe that I have an appropriate level of expertise to confirm that it is of an acceptable scientific standard.
